# The Treatment of Chronic Bright's Disease

**Published:** 1901-12-21

**Authors:** 


### Hospital Clinics and Medical Progress.

### THE TREATMENT OF CHRONIC BRIGHT'S DISEASE.

Christmas is a season when attention may with great propriety be directed to the subject of Bright's disease, for the time of year which to so many is a period of rejoicing and merrymaking is often one of much trial and many dangers to the patient whose kidneys are defective. In essence no doubt chronic interstitial nephritis is a slowly progressive disorder the steady advance of which is but little altered for good or ill by any particular thing a man may do, or any particular vicissitude to which he may be exposed. But this disease is, above almost all others, one the immediate gravity of which is influenced by the intercurrent maladies with which it may happen from time to time to be mixed up. A chill by which greater strain is thrown upon the kidney, a little indigestion by which abnormal products of gastric fermentation are thrown into the circulation, from which they have to be eliminated by the kidneys, a little over-feeding by which the amount of nitrogenous excreta are increased, a little over-exertion by which the already hypertrophied heart becomes dilated and overstrained ; any of these things may change a case, which really is one of chronic and almost imperceptible degeneration, into one of acute disease demanding the most careful treatment if the patient is to be saved from an immediately impending catastrophe ; and all of these are things which at and about Christmas time are peculiarly liable to happen. In dealing with a case of chronic interstitial nephritis, or chronic Bright's disease, we cannot do better than follow the advice given by Dr. Hale White.[1] The great essential, he says, is to relieve the kidneys as much as possible, hence the skin must be kept warm. Patients suffering from chronic interstitial nephritis often get tubular nephritis also as a result of chill, and this may be the cause of rapid death. Hence the patient must always wear flannel or something equally warm and absorbent next the skin. Not only must he never get cold but he must never get so warm as to moisten the skin and so risk a chill. A wintry east wind may easily prove fatal, in fact it does every winter prove fatal to many, so that if it be possible the patient should winter in Egypt. Moderate exercise, so that no chill follows it, is good. If fairly well, a Turkish bath is often useful, but the hotter rooms should be avoided, no cold plunge should be taken, and the patient should dress quickly. At home baths should be warm or tepid, never cold. They may be taken daily or every other day, and rough towelling is often desirable. Everything must be done to keep the skin in a clean and active condition. Whatever society may say, low dresses must not be worn, and hot and crowded assemblies must be avoided. The risk of after-chill is too great, and even when patients go to Egypt they must be careful about the nights, for in comparison with the days the nights in Egypt are cold. Then one must remember that exercise involves excretion, and that in this disease excretion is a difficulty, so that although gentle exercise is good, the patient must never allow himself to become tired, and if he should happen to make himself perspire, he should take a warm bath and change his clothes. The bowels must be kept open every day, or even twice a day, and best by taking some mineral water containing sulphate of magnesia on rising in the morning. Mental occupation, sufficient to maintain an interest in life, is useful, but never enough to exhaust or even tire, and mental anxiety is especially to be avoided. As to diet, there has been much that is fantastic and without real meaning or excuse in what has from time to time been recommended in chronic Bright's disease. "If any method at all underlies many of the dietetic directions given to patients with Bright's disease, it is that those who order the diets try by means of dieting to diminish the risk of uræmia. But we have no certain knowledge what is the poison which leads to uræmia," and there is no series of cases published to show that any particular diet is more likely than others to produce this accident. The great guiding principle in dieting a case of Bright's disease is to give such food as will keep the patient in the best general health, usually such a combination of fats, carbo-hydrates, and proteids, as is generally desirable in health : variations being introduced to meet any digestive or other peculiarity in the case. To a certain extent the pulse is a guide to the diet. If the pulse tension is low and the patient is suffering from cardiac weakness, then meat and a moderate amount of beef tea will almost certainly improve his health. One must not, however, overdose a patient with meat extracts. On the other hand, if the pulse tension is high and the heart is hypertrophied there is much danger of cerebral hæmorrhage, and meat extracts, soups, and meat itself, are all bad. Many cases of cerebral hæmorrhage have occurred after a big meal. Such patients must have frequent small meals consisting of milk, butter, and farinaceous food, with vegetables and fruit, but only a little meat and no soup at dinner. He should also have no alcohol. On the other hand a patient with a weak pulse will be all the better for a little whisky. Let the albuminuria alone. The passage of albumin is in itself of but little harm to the patient, and its amount in no way determines the prognosis. Patients with this disease are often thirsty, and they may drink water, warm water, flavoured or not with lemon or lime juice, or Imperial drink sweetened with sacharine. No alcohol except with meals, and then only whisky.

Now for the complications. In uræmia the first thing to do is, without doubt, to see that the bowels are opened. If the symptoms are slight an abundant saline purge every morning may be enough, but if more severe 30 or 40 grains of compound jalap or scammony powder should be given at once, and if this does not act soon two to four grains of compound elaterium powder should be administered. If the patient is unconscious, one or two drops of croton oil may be mixed up with a little butter and placed at the back of the tongue so as to be reflexly swallowed. To get the skin to act pilocarpin may be given subcutaneously. One-sixth of a grain is generally enough. It is very important not to give too much, for it is a powerful cardiac depressant, and, moreover, it may increase bronchial secretion to such an extent as to lead to dangerous symptoms. After administering pilocarpin the patient must be wrapped in hot blankets to encourage action of the skin, and these blankets must be changed when wet with sweat. The object is not merely to cause sweating, but to get rid of the poison, whatever it may be. Bleeding may be beneficial in severe cases, especially when there are convulsions. Diarrhœa and vomiting must be regarded as in a manner eliminative, and not to be checked unless exhausting. For the treatment of headache a purgative, a hot bath, a dark room, and a cup of strong warm tea will be found useful, while caffeine and nitro-glycerine may also be of service. When uræmic convulsions are severe and other remedies, including bleeding, have done no good, the best drug is morphine, a sixth of a grain being administered subcutaneously. Chloroform also is of service in the same circumstances. In sleeplessness 25 grains of chloramide dissolved in a little brandy or whisky may be taken an hour or two before bedtime, or trional or sulphonal may be tried. Dr. Hale White makes the practical suggestion that if these drugs are given in tablets these should be crushed before administration, as they dissolve very slowly in the gastro-intestinal tract. Patients with cardiac weakness are often benefited by a mixture of digitalis caffeine and nux vomica, while on the other hand when pulse tension is high, nitro-glycerine or iodide of potassium, in addition to care in keeping the bowels open, may be useful. If the sufferer with chronic Bright's disease cannot winter in Egypt, and be careful even there, it seems evident that his best way of getting safely through this festive season is to eschew the merriment thereof and keep himself warm and quiet.

[1] Practitioner, Dec., 1901.

